# In This Issue

**Published:** 1995

## ALCOHOL-RELATED COGNITIVE IMPAIRMENTS: AN OVERVIEW OF HOW ALCOHOLISM MAY AFFECT THE WORKINGS OF THE BRAIN

Cognition is best defined as the acquiring, storing, retrieving, and use of knowledge. Cognitive impairment has long been linked with the chronic abuse of alcohol. Because alcoholics express many types and degrees of cognitive deficits, however, defining a deficit pattern has been a complex and inexact task. Drs. Denise L. Evert and Marlene Oscar-Berman describe theoretical models developed by scientists to better characterize the cognitive impairments observed in alcoholics. The authors also discuss the experimental evidence supporting and challenging each model. (pp. 89–96)

## ASSESSING COGNITIVE IMPAIRMENT

The fact that excessive alcohol consumption can impair cognitive functioning is well documented in the alcohol literature. Yet the descriptions of how impairment has been assessed often are inadequate. Dr. Sara Jo Nixon reviews a sampling of the plethora of assessment tools currently used to evaluate alcohol’s impact on cognitive functioning. These tools are based on different testing principles: Some instruments allow assessment of overall intelligence and cognitive functioning, whereas others analyze only specific cognitive domains. By choosing the appropriate assessment tool for each patient and research situation, researchers can gain a clearer understanding of alcohol’s effects on the brain. (pp. 97–103)

## WHEN ALCOHOLISM AFFECTS MEMORY FUNCTIONS: MRI OF THE BRAIN

Despite serious memory deficits, alcoholic amnesics may perform normally on certain tests that require learning, although they may not be able to recall the information learned. Because of this phenomenon, researchers have postulated that independent memory mechanisms may exist for implicit (unconscious) and explicit (conscious) memory. Magnetic resonance imaging (MRI) makes it possible to examine directly the relationship between brain abnormalities and memory impairment. MRI studies show that damage to specific brain structures is associated with particular memory defects. Researchers Drs. Terry L. Jernigan and Arne L. Ostergaard discuss results of recent MRI studies which suggest that the observed distinction between implicit and explicit memory does not require the existence of independent memory mechanisms. (pp. 104–107)

## EVENT-RELATED POTENTIALS AND COGNITIVE FUNCTION IN ALCOHOLICS

Subtle cognitive deficits can be studied using event-related potentials (ERP). These brain waves are elicited in response to sensory stimuli to provide an immediate record of the brain activity associated with information processing. Drs. Bernice

Porjesz and Henri Begleiter discuss the abnormal ERP patterns found in alcoholics. Some ERP abnormalities may be consequences of heavy drinking, whereas anomalies that are not altered with prolonged abstinence may antecede the development of alcoholism. The authors also review evidence indicating that ERP characteristics are themselves genetically determined. (pp. 108–112)

## ALCOHOL-RELATED THIAMINE DEFICIENCY: IMPACT ON COGNITIVE AND MEMORY FUNCTIONING

Wernicke-Korsakoff syndrome (WKS) is one of the best recognized and identified neurologic disorders associated with chronic alcohol abuse. In addition to other cognitive deficits, WKS patients are unable to learn and form new memories and to remember past events. Dr. Philip J. Langlais reviews studies in human patients and laboratory animals which suggest that inadequate levels of vitamin B_1_ (thiamine) may contribute to the brain damage and cognitive impairments characteristic of WKS. Dr. Langlais then describes the link between alcohol, thiamine deficiency, and the disruption of essential brain regions. (pp. 113–121)

## THE ROLE OF LIVER DISEASE IN ALCOHOL-INDUCED COGNITIVE DEFECTS

Liver cirrhosis, a consequence of chronic alcohol abuse, prevents the liver from effectively eliminating toxic metabolic products from the bloodstream. Consequently, toxic substances reach the brain, where they can disrupt normal brain functioning. The most prevalent brain disorder resulting from liver dysfunction is portal-systemic en-cephalopathy (PSE). PSE progresses from subtle signs, such as anxiety and depression, to severe complications, such as hepatic coma. As Dr. Roger Butterworth describes, the exact correlation between cirrhosis, toxic substances (such as ammonia), and cognitive and motor dysfunctions stemming from PSE still are under intense investigation. (pp. 122–129)

## ALCOHOL-INDUCED SLEEPINESS AND MEMORY FUNCTION

Alcohol-induced memory impairments have received much attention over the years. Little is known, however, about the behavioral and neurobiological mechanisms behind such alcohol-associated impairment. It is well known that alcohol consumption has a sedating effect (i.e., leads to sleepiness). Does the sedating effect of alcohol have a role in memory impairment? Based on studies comparing the sedative and memory-impairing effects of alcohol with the memory-impairing effects of sedative drugs, Drs. Timothy Roehrs and Thomas Roth propose that alcohol-induced sleepiness does indeed contribute to memory impairment. Such a correlation could mean that any condition that induces sleepiness may increase the risk of memory impairment after alcohol consumption. (pp. 130–135)

## ALCOHOL AND THE CEREBELLUM: EFFECTS ON BALANCE, MOTOR COORDINATION, AND COGNITION

In addition to its part in motor coordination, the cerebellum may play a role in the acquisition of motor skills and the cognitive processes that control movement. Dr. Edith V. Sullivan and colleagues are investigating a possible correlation between cerebellar structural damage and cognitive impairment in alcoholics. The reversibility of alcohol-related abnormalities in cerebellar structure and function also is under investigation. Findings from such research may provide knowledge to guide future rehabilitation efforts. (pp. 138–141)

## COGNITIVE IMPAIRMENT IN CHILDREN OF ALCOHOLICS

Children of alcoholics (COA’s), particularly sons of male alcoholics, frequently have deficits in verbal skills, abstract thinking, goal-directed planning, and other cognitive functions. In addition, COA’s have a higher incidence of behavioral problems and a greater risk of becoming alcoholic themselves than do children of nonalcoholics. Dr. Robert O. Pihl and Kenneth R. Bruce present an information-processing model that helps to explain how cognitive deficits may contribute to the COA’s behavioral problems and risk for alcoholism. (pp. 142–147)

## RECOVERY OF COGNITIVE FUNCTIONING IN ALCOHOLICS: THE RELATIONSHIP TO TREATMENT

An encouraging observation is that all impaired alcoholics show some degree of recovery of cognitive and behavioral functioning once alcohol use is discontinued, according to Dr. Mark S. Goldman. Moreover, recovery can be facilitated using methods such as repeated mental exercises. Dr. Goldman reviews the nature and course of recovery from alcohol-related deficits and offers strategies for enhancing this process. He also discusses growing evidence that supports an association between cognitive functioning and treatment outcome. (pp. 148–154)

